# Differential serum dynamics of apelin, elabela, and angiotensinogen in CKD and haemodialysis: insights into peptide modulation across the dialysis procedure

**DOI:** 10.1080/0886022X.2026.2646404

**Published:** 2026-03-26

**Authors:** Natalia Maria Serwin, Elżbieta Cecerska-Heryć, Bartłomiej Grygorcewicz, Mateusz Cejko, Adrianna Jerzyk, Kacper Ćwikła, Emilia Spajzer, Aleksandra Manelska, Paulina Woźniak, Aleksandra Gomółka, Aleksandra Cader-Ptak, Katarzyna Bąk, Marcin Lisak, Martyna Opara-Bajerowicz, Bartosz Wojciuk, Mariusz Suwała, Karol Serwin, Magda Wiśniewska, Barbara Dołęgowska

**Affiliations:** a Department of Laboratory Medicine, Pomeranian Medical University in Szczecin, Poland; b Department of Genomics and Forensic Genetics, Pomeranian Medical University in Szczecin, Poland; c Faculty of Pharmacy, Biotechnology, and Laboratory Medicine, Pomeranian Medical University in Szczecin, Poland; d Department of Nephrology, Transplantology, and Internal Medicine, Pomeranian Medical University in Szczecin, Poland; e Department of Immunological Diagnostics, Faculty of Medicine, Pomeranian Medical University in Szczecin, Poland; f Department of Periodontology, Pomeranian Medical University in Szczecin, Poland; g Department of Infectious, Tropical Diseases, and Acquired Immunodeficiency, Pomeranian Medical University in Szczecin, Poland

**Keywords:** Apelin, elabela, angiotensinogen, hemodialysis, chronic kidney disease

## Abstract

**Background:**

Emerging evidence suggests that apelinergic peptides and components of the renin–angiotensin system (RAS) are key regulators of cardiovascular homeostasis and fluid balance, both disrupted in chronic kidney disease (CKD). This study aimed to assess serum levels of apelin, elabela, and angiotensinogen across the CKD spectrum and evaluate their acute modulation during a single hemodialysis (HD) session. Serum samples from healthy controls (*n* = 21), non-dialysis CKD patients (*n* = 21), and HD patients (*n* = 44) were analyzed using high-sensitivity immunoassays. In HD patients, matched samples were collected before and after dialysis.

**Methods:**

Apelin levels were significantly lower in CKD compared to controls but elevated in HD patients prior to treatment. No significant change occurred post-dialysis, suggesting minimal dialytic clearance or rapid compensation. In contrast, elabela was markedly reduced pre-dialysis but increased post-dialysis in most patients, indicating dynamic regulation or partial removal. Angiotensinogen was elevated before HD and declined significantly after.

**Results:**

Correlations with estimated glomerular filtration rate (eGFR) were modest and not significant after adjusting for group membership, indicating that group stratification primarily drove associations. Linear regression showed that reductions in angiotensinogen correlated inversely with changes in apelin, while higher uric acid and urea predicted post-dialysis increases in elabela and suppression of angiotensinogen, respectively.

**Conclusion:**

These findings highlight distinct, peptide-specific responses to HD and support a context-dependent interplay between uremic burden, peptide systems, and dialysis-induced shifts. Apelinergic and RAS peptides may represent promising biomarkers of intradialytic cardiovascular modulation. Further studies are warranted to explore their prognostic and therapeutic implications in CKD.

## Graphical abstract

**Figure:**
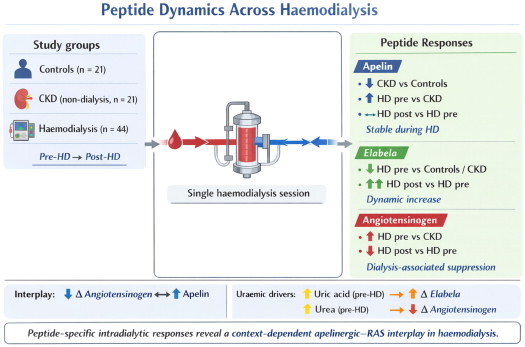

## Introduction

Chronic kidney disease (CKD) leads to progressive disruptions in cardiovascular and fluid homeostasis. These changes are closely linked to alterations in peptide-based regulatory systems, including the RAAS and the apelinergic axis. Both systems are involved in blood pressure control, sodium handling, and vascular integrity – functions frequently compromised in CKD. Growing evidence suggests that these peptides may also respond dynamically to interventions such as hemodialysis.

The renin–angiotensin–aldosterone system (RAAS) is a key hormonal axis regulating blood pressure, sodium balance, and extracellular fluid volume. Its initiating substrate, angiotensinogen (AGT), is essential for generating all downstream angiotensin peptides. Understanding AGT synthesis and distribution is critical for interpreting RAAS activity in systemic and local contexts, particularly in hypertension, chronic kidney disease (CKD), and heart failure. The liver is the dominant source of circulating AGT, producing over 90% of plasma levels under physiological conditions [1,2]. AGT is secreted constitutively by hepatocytes and regulated by hormones and inflammatory stimuli. Liver-specific deletion of the AGT gene in murine models results in near-complete loss of systemic Ang II, confirming the liver’s central role in the systemic RAAS [1]. Although AGT is also expressed in adipose tissue, kidney, brain, and vasculature, these extrahepatic sources primarily contribute to local (paracrine/autocrine) RAAS activity. In obesity, for example, adipose-derived AGT has been implicated in vascular and metabolic dysfunction [3]. However, these tissues do not significantly affect circulating AGT concentrations under normal conditions [1].

The intrarenal RAAS is a compartmentalized extension of the RAAS within the kidney. While it largely depends on filtered hepatic AGT as its substrate, AGT is also synthesized locally by proximal tubule epithelial cells and, to a lesser extent, by glomerular and collecting duct cells [4]. This local system can be independently activated, particularly in pathological states such as salt-sensitive hypertension or diabetic nephropathy [4,5]. Renin is classically produced by juxtaglomerular cells, but is also expressed in collecting duct principal cells, particularly under Ang II–dependent conditions [6]. ACE and ACE2 are distributed along the nephron, enabling local conversion of Ang I to Ang II and Ang II to Ang- [1–7], respectively [4,7]. Locally produced Ang II acts on AT_1_ receptors in tubular epithelium and vasculature, promoting sodium reabsorption, efferent arteriole constriction, and regulation of glomerular filtration rate (GFR). Notably, intrarenal Ang II levels may remain elevated even when systemic RAAS activity is suppressed, indicating functional autonomy [4,5].

Parallel to the RAAS, the apelinergic system – comprising the G protein–coupled apelin receptor (APJ) and its two known endogenous ligands, apelin and elabela (ELA) – has emerged as a critical modulator of cardiovascular and renal physiology. Apelin acts as a vasodilator, inotrope, and diuretic peptide with antagonistic effects on the RAAS, whereas ELA has been identified as a key regulator of early nephrogenesis and adult fluid balance [8,9]. Both peptides are increasingly recognized for their protective roles in kidney injury, fibrosis, and cardiovascular remodeling. The circulating levels of apelin and ELA appear to be differentially regulated in CKD. Apelin is often found to be elevated in moderate-to-severe renal impairment, possibly as a compensatory response to increased vascular resistance and RAAS activation [10]. In contrast, ELA concentrations decline progressively with decreasing glomerular filtration rate (GFR), suggesting a link to nephron mass or renal biosynthetic capacity [11]. These observations imply that apelin and ELA may have distinct biological trajectories in CKD pathophysiology and differing responses to dialysis-induced shifts in hemodynamics and inflammatory burden.

Despite growing interest in apelinergic signaling, few studies have comprehensively assessed the serum dynamics of apelin and ELA across the dialysis procedure. Furthermore, the simultaneous evaluation of AGT, apelin, and ELA in this setting remains scarce. Understanding the interplay between these peptide systems may yield novel insights into the vascular, inflammatory, and endocrine changes accompanying hemodialysis and may inform future biomarker or therapeutic strategies. Therefore, this study aimed to characterize and compare the serum dynamics of three peptide modulators – apelin, elabela, and angiotensinogen – in patients with chronic kidney disease (CKD) and those undergoing maintenance hemodialysis (HD). By evaluating pre- and post-dialysis serum concentrations, we sought to elucidate the impact of the dialysis procedure on the circulating levels of these peptides and to explore their potential role as biomarkers reflecting fluid status, vascular regulation, and neurohormonal activity. To our knowledge, this is the first study to simultaneously assess these three interrelated peptides across the dialysis session, providing novel insight into their differential regulation in advanced kidney disease.

## Materials and methods

### Participants

This was an observational, cross-sectional study with an embedded pre–post sampling component in the hemodialysis (HD) group. A total of 86 adults were recruited consecutively from the Department of Nephrology, Transplantology and Internal Medicine of the Pomeranian Medical University Hospital (USK2) in Szczecin between March 2023 and February 2025. The cohort included 21 healthy controls, 21 patients with non-dialysis chronic kidney disease (CKD), and 44 patients undergoing maintenance hemodialysis.

Eligibility criteria included age ≥18 years and written informed consent. Control participants had normal kidney function on routine testing and no known renal, cardiovascular or systemic inflammatory disease. CKD participants had a confirmed diagnosis of CKD (KDIGO stages G3–G5) according to established clinical guidelines. HD patients had been receiving thrice-weekly maintenance hemodialysis for at least three months. Paired blood samples in the HD group were collected immediately before and after a routine mid-week dialysis session; only stable sessions without acute intradialytic complications were included.

Hemodialysis was performed using low-flux or high-flux dialyzers, with dialysis dose and membrane type individually adjusted according to body weight and monthly adequacy assessment (Kt/V). A subset of patients was treated with haemodiafiltration as part of routine clinical practice.

Comorbidity profiles reflected the typical spectrum of advanced kidney disease. The most common conditions were hypertension (38.6%), glomerulonephritides (15.9%), diabetes mellitus (11.4%), obstructive uropathies including congenital abnormalities and stone-related obstruction (11.4%), and autosomal dominant polycystic kidney disease (9.1%). Less frequent etiologies included atherosclerotic nephropathy, thrombotic microangiopathy, cardio–renal syndrome and congenital syndromes (9.1% combined). Background pharmacotherapy was heterogeneous and represented standard clinical management rather than protocolised treatment. Most CKD and HD patients received combinations of renin–angiotensin system inhibitors, beta-blockers, calcium-channel blockers, loop diuretics and statins, while individuals with diabetes were treated with insulin and/or oral antidiabetic agents. Because medical therapy varied substantially between individuals, medications were not analyzed as stratified covariates.

The study adhered to STROBE recommendations for observational research, and all demographic, clinical and biochemical data were obtained from structured interviews and electronic medical records.

Demographic data included age, sex and body mass index (BMI). Clinical variables comprised primary kidney disease, comorbidities, blood pressure, and chronic medications. In hemodialysis patients, dialysis duration, treatment schedule, dialysis adequacy (Kt/V), ultrafiltration volume and interdialytic weight gain were obtained from routine session reports.

Laboratory assessments were derived from fasting blood samples in the control and CKD groups and from pre- and post-dialysis samples in the HD group. The following parameters were recorded according to routine clinical practice: serum creatinine, urea, electrolytes (sodium, potassium), bicarbonate, albumin, C-reactive protein, fasting glucose, lipid profile, complete blood count and uric acid. For the purposes of this study, additional measurements included serum apelin, elabela (ELA) and angiotensinogen (AGT), determined using ELISA-based assays as described in the analytical section. Estimated glomerular filtration rate (eGFR) was calculated using the CKD-EPI formula.

All data were retrieved from structured clinical interviews, electronic medical records and dialysis unit software, ensuring completeness and consistency across study groups.

### Material

Fasting blood samples were collected in the morning from the antecubital vein into two separate tubes: one containing a clot activator for serum collection. All samples were allowed to rest at room temperature for 30 min, followed by centrifugation at 1000 × g for 10 min at room temperature, and transferred into clean tubes and stored frozen until analysis.

### Methods

Serum concentrations of Elabela (ELA) were determined using a commercially available Human ELA ELISA Kit (Catalog No. ELK0737, ELK Biotechnology, USA). The assay is based on a competitive inhibition enzyme immunoassay technique (sensitivity of 0.61 ng/mL, with a quantifiable detection range from 1.57 to 100 ng/mL). The manufacturer states that the assay demonstrates no significant cross-reactivity or interference with Human ELA analogues. The serum concentration of Apelin (AP) in serum supernatants was measured using the Human Apelin ELISA Kit (Catalog No. E2014Hu, Shanghai Korain Biotech Co., Ltd., China) according to the manufacturer’s instructions (detection range of 7–1500 ng/L and a sensitivity of 3.47 ng/L) According to the manufacturer’s documentation, the assay is validated for serum and EDTA plasma and does not report cross-reactivity with ELA or other APJ ligands.

Human Angiotensinogen (AGT) levels in serum were measured using a commercial sandwich ELISA kit (Catalog No. BPE162, Human Angiotensinogen, AGT Primacu^™^ ELISA Kit, Shanghai Korain Biotech Co., Ltd., China), according to the manufacturer’s protocol (detection range of 312.5–20,000 pg/mL and a sensitivity of 148.81 pg/mL). The manufacturer indicates that the assay does not exhibit measurable interference from renin, angiotensin I, angiotensin II, or related RAS components.

### Statistical analysis

All analyses were performed in R (v4.3.2). Biomarker concentrations were log10-transformed before testing. Between-group differences were assessed using Wilcoxon tests: rank-sum for independent groups (Control, CKD, HD_pre) and paired signed-rank tests for pre- and post-HD comparisons. False discovery rate (FDR) correction was applied. Associations with eGFR were evaluated using Spearman and partial correlations (adjusted for group). For selected predictor–outcome pairs, linear regression models were fitted, reporting β coefficients, R^2^ values, and p-values. All linear models underwent standard diagnostic evaluation, including inspection of residual plots, assessment of homoscedasticity and linearity, and identification of influential observations. No violations of model assumptions were observed that would preclude interpretation. Given the sample size, the regression analyses were considered exploratory and were not intended for predictive modeling.

## Results

**Figure 1.**
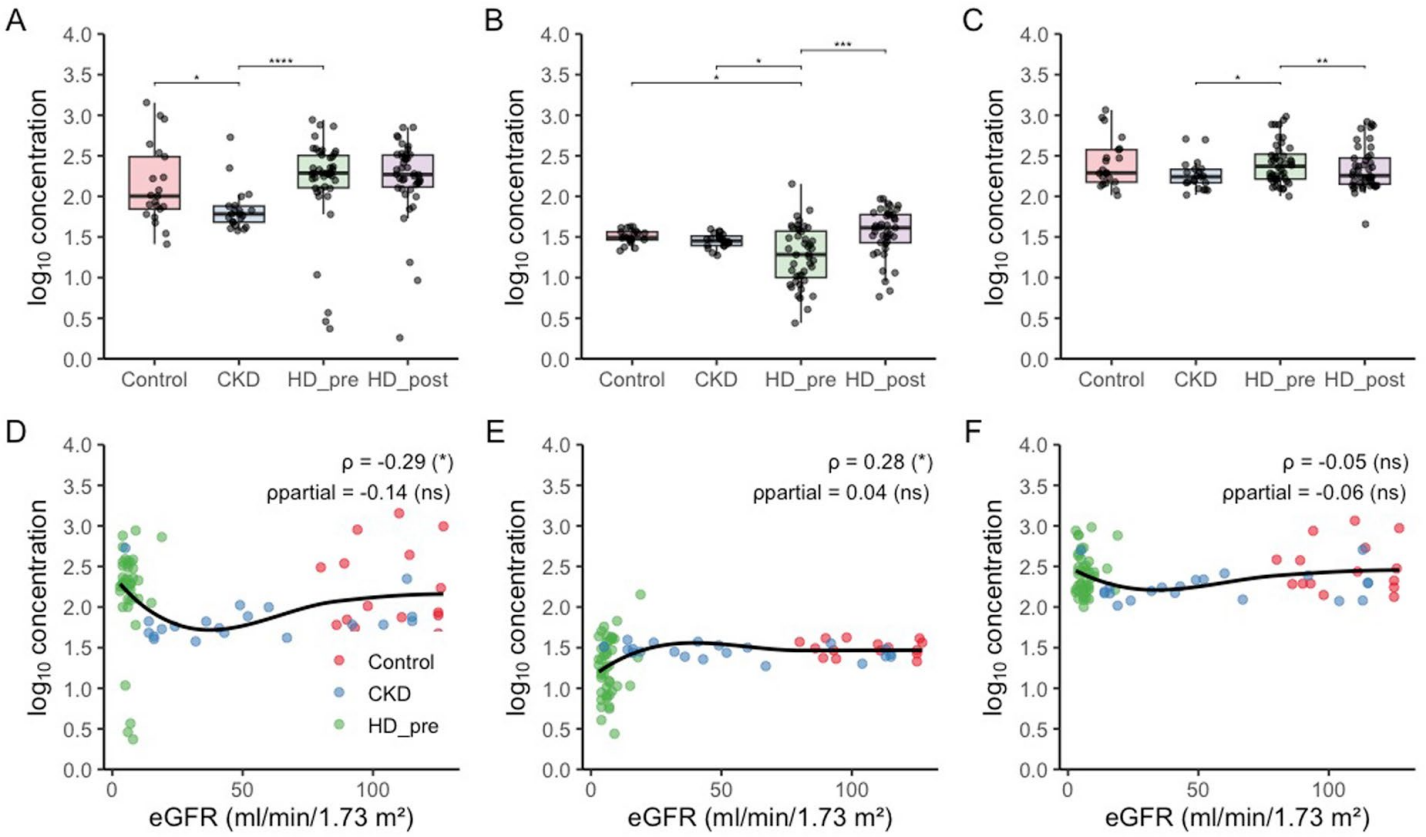
Box-plots (panels a–c) show log_10_ concentrations of apelin, elabela and angiotensinogen in controls, non-dialysis CKD, and hemodialysis patients sampled immediately before (HD pre) and after (HD post) a session; boxes indicate median and IQR, whiskers extend to 1.5 × IQR, points are individual subjects, and horizontal brackets mark Wilcoxon contrasts (rank-sum for independent groups, signed-rank for the paired HD pre–HD post comparison) with significance codes **** *p* < 0.0001, *** *p* < 0.001, ** *p* < 0.01, * *p* < 0.05. Scatter-plots (panels d–f) relate baseline biomarker levels to eGFR; colored symbols denote groups, black curves are LOESS fits, and in-panel text reports the cohort-wide Spearman coefficient (ρ) and the partial Spearman controlling for group (ρ_partial), illustrating that the raw inverse biomarker–eGFR association is largely accounted for by between-group stratification rather than within-group variation. Note that confidence intervals are not applicable to box-plot displays, as whiskers represent data variability (IQR ± 1.5 × IQR) rather than 95% CI.

Detailed descriptive data of the analyzed groups are shown in Table 1. Among all participants (*n* = 86), the median age was 67.0 years (IQR 52.0–73.5), 27.3% were female, and the median body mass index (BMI) was 27.5 kg/m^2^ (IQR 22.6–31.9). In serum, apelin was lower in CKD than in controls (median 60.7 ng ml^−1^ vs 100.8 ng ml^−1^, *p* = 0.0128) and higher in patients sampled immediately before hemodialysis (HD pre) than in CKD (193.6 ng ml^−1^ vs 60.7 ng ml^−1^, *p* = 2.6 × 10^−5^); no difference was detected across the dialysis procedure (HD pre vs HD post, *p* = 1.0). ELA was lower in HD pre than in both controls (19.2 ng ml^−1^ vs 31.0 ng ml^−1^, *p* = 0.0105) and CKD (19.2 ng ml^−1^ vs 28.3 ng ml^−1^, *p* = 0.0487), but increased after dialysis (41.0 ng ml^−1^ vs 19.2 ng ml^−1^, *p* = 7.6 × 10^−4^). AGT differed between CKD and HD pre (174 ng ml^−1^ vs 235 ng ml^−1^, *p* = 0.0181) and declined post-dialysis (180 ng ml^−1^ vs 235 ng ml^−1^, *p* = 0.0043) (Figure 1, panels a–c); all other pairwise contrasts were non-significant. Baseline correlations with estimated glomerular filtration rate (Figure 1, panels d–f) were modest: apelin ρ = −0.29 (*p* = 0.011), and elabela ρ = 0.28 (*p* = 0.013), while angiotensinogen showed no association (ρ = −0.05, *p* = 0.66). After adjustment for group membership, none of the correlations remained significant (apelin ρ_partial = −0.14, *p* = 0.22; ELA ρ_partial = 0.04, *p* = 0.70; AGT ρ_partial = −0.06, *p* = 0.60).

**Table 1.** Descriptive statistics of the analyzed populations.

Parameter	Control	CKD	HD_pre	HD_post
Age [years]	33.0 [19.0–45.0] (*n* = 21)	56.0 [41.0–77.0] (*n* = 21)	67.0 [52.0–73.5] (*n* = 43)	67.0 [52.0–73.5] (*n* = 43)
Sex [Female/male]	F: 12 (57.1%), M: 9 (42.9%)	F: 13 (61.9%), M: 8 (38.1%)	M: 30 (68.2%), F: 14 (31.8%)	M: 30 (68.2%), F: 14 (31.8%)
eGFR [mL/min/1.73 m²]	110.0 [91.5–125.0] (*n* = 15)	43.0 [19.0–92.0] (*n* = 21)	6.0 [4.0–8.0] (*n* = 44)	NaN
Serum urea [mg/dl]	NaN	NaN	115.0 [91.8–143.0] (*n* = 44)	38.5 [30.0–50.0] (*n* = 44)
Serum uric acid [mg/dl]	NaN	NaN	5.7 [5.1–6.6] (*n* = 44)	NaN
ALAT [U/l]	NaN	NaN	12.0 [9.0–17.0] (*n* = 44)	12.0 [9.0–17.0] (*n* = 44)
ALP [U/l]	NaN	NaN	84.0 [62.0–101.2] (*n* = 44)	84.0 [62.0–101.2] (*n* = 44)
Triglycerides [mg/dl]	126.7 [118.7–135.9] (*n* = 21)	136.7 [135.1–139.5] (*n* = 18)	108.5 [69.0–157.0] (*n* = 44)	108.5 [69.0–157.0] (*n* = 44)
Cholesterol [mg/dl]	185.6 [179.5–190.4] (*n* = 21)	191.1 [182.6–200.1] (*n* = 18)	137.5 [117.8–166.5] (*n* = 44)	137.5 [117.8–166.5] (*n* = 44)
Height [cm]	NaN	NaN	170.0 [164.8–176.0] (*n* = 44)	170.0 [164.8–176.0] (*n* = 44)
Weight [kg]	NaN	NaN	75.5 [69.5–92.6] (*n* = 44)	75.5 [69.5–92.6] (*n* = 44)
Waist circumference [cm]	NaN	NaN	102.5 [93.0–107.8] (*n* = 22)	102.5 [93.0–107.8] (*n* = 22)
Serum creatinine [mg/dl]	0.8 [0.7–0.9] (*n* = 15)	1.5 [0.9–2.7] (*n* = 21)	8.0 [6.8–10.0] (*n* = 44)	NaN
Glucose [mg/dl]	79.5 [78.0–82.1] (*n* = 21)	85.5 [82.4–87.5] (*n* = 18)	NaN	NaN
Total protein [g/dl]	6.3 [6.0–6.6] (*n* = 21)	6.2 [5.9–6.5] (*n* = 18)	NaN	NaN
Albumin [g/dl]	4.2 [3.9–4.4] (*n* = 21)	3.8 [3.5–3.9] (*n* = 18)	NaN	NaN
Elabela [ng/ml]	31.0 [29.1–36.5] (*n* = 21)	28.3 [24.8–32.5] (*n* = 21)	19.2 [10.0–37.3] (*n* = 44)	41.0 [26.9–59.6] (*n* = 41)
Apelin [ng/ml]	100.8 [69.9–307.9] (*n* = 21)	60.7 [48.2–76.0] (*n* = 21)	193.6 [127.6–319.3] (*n* = 42)	186.0 [130.4–323.0] (*n* = 42)
Angiotensinogen [ng/ml]	194.7 [149.9–376.2] (*n* = 21)	174.4 [147.2–215.5] (*n* = 21)	234.7 [163.7–331.5] (*n* = 44)	180.5 [141.4–297.1] (*n* = 44)
BMI	NaN	NaN	27.5 [22.6–31.9] (*n* = 44)	27.5 [22.6–31.9] (*n* = 44)

Data are shown as median (lower quartile-upper quartile)-Q3). NaN – data not available; NaN indicates variables that were not routinely collected in some subgroups or were not clinically applicable. Certain measurements (e.g. waist circumference and selected laboratory parameters) were available only for hemodialysis patients as part of routine unit assessments, whereas others (e.g. post-dialysis creatinine) are not obtained or not meaningful after dialysis.

**Figure 2.**
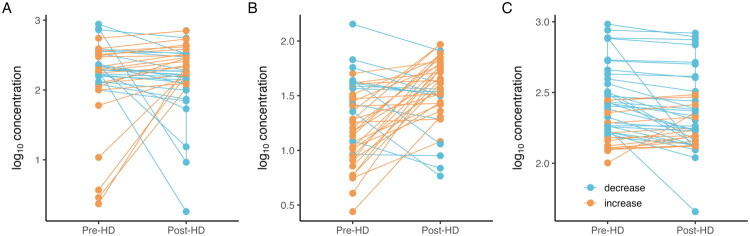
Log-transformed concentrations of apelin (panel A), elabela (panel B), and angiotensinogen (panel C) in serum of hemodialysis patients before and after a dialysis session. Each line represents an individual patient. Arrows indicate the direction of change (↑: increase, ↓: decrease) between pre- and post-dialysis timepoints.

To further evaluate the acute effect of dialysis on circulating biomarker levels, we analyzed matched pre- and post-dialysis samples from hemodialysis patients (Figure 2). ELA concentration increased significantly following the dialysis session (median 19.2 ng/ml vs 41.0 ng/ml, *p* = 7.6 × 10^−4^), with nearly all individuals showing a consistent upward trend. In contrast, AGT levels decreased in most patients (median 235 ng/ml vs 180 ng/ml, *p* = 0.0043). Apelin levels exhibited marked baseline heterogeneity but did not change significantly post-dialysis (median 193.6 ng/ml vs 190.2 ng/ml, *p* = 1.0). These directional trends suggest differential clearance or production dynamics during hemodialysis across peptide systems.

**Figure 3.**
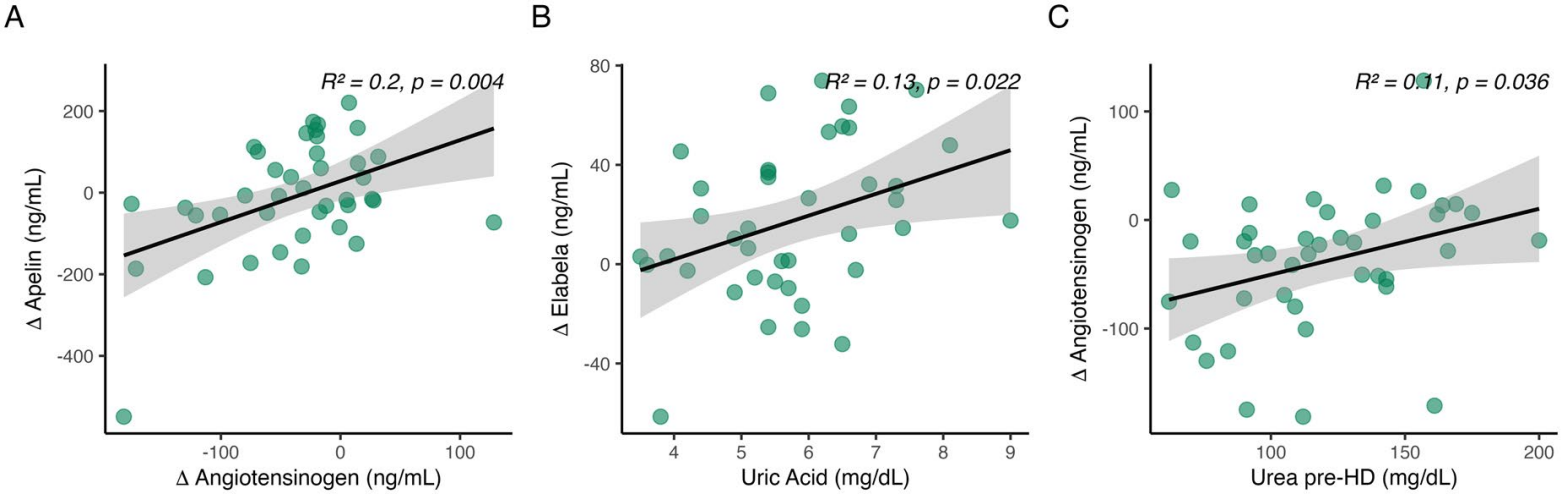
Relationships between renal biomarkers and intradialytic changes (Δ) in peptide levels. (A) ΔAngiotensinogen is negatively associated with ΔApelin (β = 1.01, 95% CI 0.35–1.67, *p* = 0.0038). (B) Higher pre-dialysis serum uric acid is positively associated with ΔElabela (β = 8.79, 95% CI 1.32–16.26, *p* = 0.022). (C) Higher pre-dialysis urea predicts a greater reduction in angiotensinogen (β = 0.61, 95% CI 0.04–1.17, *p* = 0.036). Lines represent linear regression fits with shaded 95% confidence intervals. Δ = post − pre.

Among tested predictors, a significant inverse association was observed between the reduction in serum AGT and the increase in apelin following dialysis ((β = 1.01, 95% CI 0.35–1.67, *p* = 0.0038, R^2^ = 0.20), supporting a biologically plausible link between RAA suppression and apelinergic upregulation. Furthermore, uric acid levels prior to dialysis were positively associated with post-dialysis increases in ELA (β = 8.79, 95% CI 1.32–16.26, *p* = 0.022, R^2^ = 0.13), suggesting a compensatory or stress-related mechanism. Pre-dialysis urea also significantly predicted the magnitude of AGT suppression (β = 0.61, 95% CI 0.04–1.17, *p* = 0.036, R^2^ = 0.11) indicating a role of uremic burden in RAA activation (Figure 3). Diagnostic evaluation of the additive models did not indicate violations of linear model assumptions; residuals showed no marked deviations from homoscedasticity or normality, and no single observation disproportionately influenced model estimates.

To investigate the relationship between body mass index (BMI) and the change in concentrations of selected biomarkers, we employed additive linear regression models. The primary outcome variables were the deltas (Δ) of three biomarkers: ELA (ng/mL), Apelin (ng/mL), and AGT (ng/mL). These values represent intra-individual changes in biomarker concentrations and were analyzed as continuous dependent variables. For each biomarker, we fitted an additive linear model of the form:

$$\Delta {\text{Biomarker}}_{i}=\beta_{0}+\beta_{1}\cdot {\text{BMI}}_{i}+\beta_{2}\cdot {\text{Sex}}_{i}+\varepsilon_{i}$$

where:

- ΔBiomarker_i_ denotes the change in biomarker concentration for participant *i*,
- BMI_i_ is the body mass index,
- Sex_i_ is a binary variable (coded as 0 = Female, 1 = Male),
- β_0_ is the intercept,
- β_1_ and β_2_ are regression coefficients,
- ε_i_ is the random error term.

All models were fit using the lm() function in R BMI was entered as a continuous predictor, and sex was treated as a categorical variable. Before modeling, the BMI variable was explicitly converted to numeric to avoid type coercion errors, and the sex variable was recoded into a factor with two levels (‘Female’, ‘Male’)

**Figure 4.**
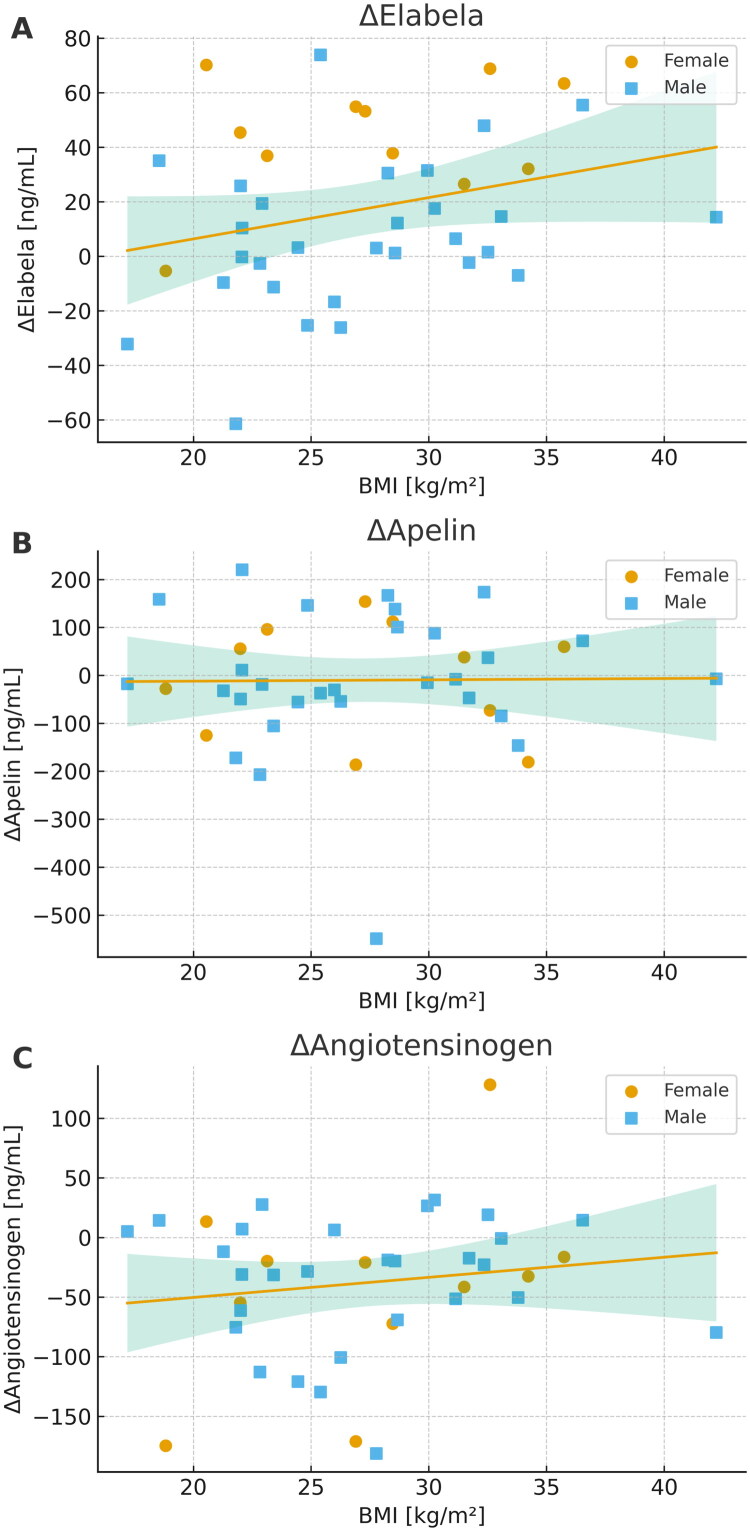
Association between body mass index (BMI) and intradialytic changes (Δ) in peptide concentrations: (A) Elabela, (B) Apelin, and (C) Angiotensinogen. Each symbol represents an individual hemodialysis patient (circles: females; squares: males). Lines depict simple linear regressions of Δ peptide levels on BMI with shaded 95% confidence intervals. In multivariable linear models including BMI and sex, BMI was positively associated with changes in elabela (R^2^ = 0.364, β_BMI = 1.47, *p* = 0.049; β_sex = –36.5, *p* < 0.001), whereas no significant associations were observed for Δapelin (R^2^ < 0.001, p_BMI = 0.948) or Δangiotensinogen (R^2^ = 0.025, p_BMI = 0.353).

We performed additive linear regression analyses to evaluate the effects of body mass index (BMI) and sex on changes in serum concentrations of the biomarkers Δelabela, Δapelin, and Δangiotensinogen (Figure 4). For Δelabela, the model explained 36.4% of the variance (*R*^2^ = 0.364). A statistically significant positive association with BMI was observed (*β* = 1.466, *p* = 0.049), suggesting that higher BMI is associated with a greater increase in Δ elabela. Additionally, male sex was significantly associated with lower levels of Δ elabela compared to females (*β* = −36.51, *p* < 0.001). In contrast, the model for Δapelin showed no meaningful explanatory power (*R*^2^ < 0.001). Neither BMI (*β* = 0.270, *p* = 0.948) nor sex (*β* = −4.18, *p* = 0.935) was significantly associated with Δapelin levels. The model for Δangiotensinogen explained only a small portion of variance (*R*^2^ = 0.025). Neither BMI (*β* = 1.697, *p* = 0.354) nor sex (*β* = 5.681, *p* = 0.801) showed a statistically significant effect.

## Discussion

This study provides novel insights into the dynamic regulation of three key peptide systems – apelin, ELA, and AGT – across the CKD spectrum and the hemodialysis (HD) procedure. Our findings highlight distinct, peptide-specific patterns that likely reflect both the uremic environment and the physiological stress of dialysis.

Apelin levels were reduced in non-dialysis CKD compared to controls, consistent with earlier studies indicating that apelin concentrations decline with progressive renal impairment, potentially due to reduced production, increased degradation, or alterations in APJ receptor regulation [10,12]. Intriguingly, in HD patients, apelin levels were elevated prior to dialysis and remained unchanged post-session. This may suggest compensatory upregulation in response to chronic RAAS activation, sympathetic overactivity, or fluid overload in the interdialytic period. The absence of a significant post-dialysis change indicates limited dialytic clearance, possibly due to apelin’s molecular size or rapid redistribution. Although the present assay quantified total circulating apelin, it did not distinguish between individual isoforms such as apelin-13, apelin-17, or apelin-36. These peptides differ in molecular weight, stability, receptor affinity, and degradation rates, and their dialyzability may not be uniform. Pooling all isoforms into a single ‘total apelin’ measurement may therefore mask subtype-specific responses to hemodialysis, potentially explaining the absence of a detectable post-dialysis change. Future studies employing isoform-specific assays or mass-spectrometry-based approaches will be necessary to determine whether particular apelin forms are preferentially affected by CKD progression or the dialysis procedure.

ELA displayed a markedly different profile. While concentrations were lowest in HD patients prior to dialysis, they rose significantly post-session. This biphasic pattern suggests that ELA is highly responsive to the hemodialysis procedure. The post-dialysis rise may reflect removal of uremic inhibitors, improved renal microperfusion, or release from peripheral tissues. These findings align with prior data showing reduced ELA in CKD and highlight its potential as a dynamic biomarker of dialysis efficacy [11,12]. Existing evidence indicates that circulating ELA levels are generally reduced in chronic kidney disease and end-stage renal disease, reflecting the kidney’s role as a major site of ELA expression. Levels in peritoneal dialysis patients appear to depend largely on residual renal function and dialysis vintage [12,13]. Unlike endothelin-1, which typically increases after hemodialysis, ELA concentrations in advanced kidney disease are already markedly diminished, and hemodialysis itself is not expected to substantially clear this peptide, given that it removes primarily small, water-soluble uremic toxins [13–15]. The mechanisms underlying acute post-HD shifts in ELA therefore remain incompletely defined. Improved renal perfusion after ultrafiltration is unlikely to be sufficient, on its own, to generate a systemic rise in ELA within the short post-dialysis interval. A more plausible explanation is transient modulation or release from vascular or renal tissues in response to the hemodynamic and endothelial stresses inherent to the dialysis procedure, including rapid volume shifts and blood pressure fluctuations. These physiological perturbations are known to influence several vasoactive peptide systems and may similarly affect ELA [16,17]. Overall, current evidence suggests that ELA dynamics are closely linked to renal function and cardiovascular integrity, and that acute post-HD changes likely reflect a complex interaction between underlying disease severity, endothelial responsiveness, and the procedural effects of dialysis on fluid and vascular homeostasis [18,19].

AGT levels were highest in HD patients before dialysis and declined significantly after the session. This decline could be attributed to several mechanisms, including volume removal, decreased inflammatory stimuli, or partial clearance through high-flux dialysis membranes. Importantly, we observed an inverse correlation between changes in AGT and apelin, which may reflect the known physiological antagonism between the RAAS and the apelinergic system. Prior studies have shown that apelin and elabela exert vasodilatory, natriuretic, and anti-fibrotic effects that counterbalance RAAS-mediated vasoconstriction and sodium retention [8,9]. Although the inverse post-dialysis relationship between AGT and apelin is consistent with the well-described antagonistic interplay between the RAAS and apelinergic systems, the present study did not assess downstream RAAS mediators, receptor expression or intracellular signaling. As such, the observed association should be interpreted as physiologically plausible but not mechanistically confirmed. Dedicated mechanistic studies will be required to validate this interaction. Moreover, elevated pre-dialysis urea and uric acid were associated with greater AGT suppression and ELA increase, respectively, supporting the concept of solute-driven modulation of these pathways. Although these findings are observational, they suggest that accumulation of specific uremic solutes may influence the balance between RAAS and apelinergic signaling during dialysis. Such solute–peptide interactions have been proposed in experimental settings, and our results provide preliminary clinical support for this possibility. At the same time, the present data cannot distinguish direct regulatory effects from broader metabolic or hemodynamic correlates, and targeted mechanistic studies will be required to clarify the underlying drivers.

Comparison with existing literature reveals substantial variability in reported peptide concentrations, likely due to differences in assay platforms, patient characteristics, and dialysis protocols. Nevertheless, our findings are directionally consistent with prior studies showing reduced ELA and variable apelin levels in advanced CKD [10,12]. Clinically, these data underscore the relevance of apelinergic and RAAS peptides as biomarkers of fluid status, uremic burden, and dialysis response. The pronounced intradialytic changes in ELA and AGT may offer real-time insight into patient-specific physiological shifts, with potential implications for volume management and cardiovascular risk stratification. Future studies should focus on serial measurements over the dialysis week, correlations with hemodynamic parameters, and the potential therapeutic modulation of these axes.

In the regression analysis incorporating body mass index (BMI) and sex, only changes in elabela concentration (ΔELA) were significantly associated with these variables. Higher BMI was linked to a greater post-dialysis increase in ELA, suggesting a possible metabolic or hormonal influence on the apelinergic system. Previous studies have indicated that ELA expression may be modulated by inflammation and oxidative stress associated with obesity and insulin resistance [9,20]. Moreover, the observation of lower ΔELA values in male patients may reflect hormonal differences, such as the influence of testosterone on APJ receptor expression or peptide synthesis, which has been described in the context of cardiovascular function as interactions between the apelin–APJ pathway and reproductive hormone synthesis have been demonstrated in experimental models, where modulation of APJ activity alters intratesticular testosterone production [21].

No significant associations were found between BMI or sex and changes in apelin or AGT levels. This may indicate a greater physiological stability of these peptides with respect to anthropometric variables, or their regulation through alternative pathways such as neurohormonal mechanisms. Notably, AGT is primarily synthesized in the liver, and its plasma levels are less susceptible to short-term fluctuations [1,3].

In summary, apelin, ELA, and AGT exhibit differential serum kinetics in CKD and hemodialysis, reflecting their distinct regulatory pathways and responses to the uremic milieu. Their coordinated assessment may enhance our understanding of peptide-based regulation in renal disease and support the development of novel diagnostic or therapeutic strategies.

## Limitations

This study has several limitations that should be considered when interpreting the findings. The cohort size was relatively small, particularly in the non-dialysis CKD subgroup, which may have reduced the statistical power to identify more subtle associations or subgroup-specific effects. The cross-sectional nature of the CKD component and the two-point pre–post sampling scheme in the hemodialysis group limit the ability to characterize full temporal dynamics of peptide fluctuations across the entire interdialytic interval. In addition, the study did not include systematic recording of intradialytic events such as hypotension, arrhythmias, or changes in perfusion, which prevented an assessment of whether acute hemodynamic instability contributes to peptide variability. Dialysis technical factors – including membrane type, flux characteristics, and the use of haemodiafiltration – were not analyzed in a stratified manner and therefore may have introduced unmeasured variability in peptide clearance. The assays used measured total circulating peptide concentrations and did not distinguish apelin isoforms, which may differ in biological activity and clearance. A further limitation is the two-point pre–post sampling scheme used in the hemodialysis group. By design, this approach provides a net snapshot of the biochemical effect of a single dialysis session, but does not capture the full temporal profile of peptide kinetics, including potential intradialytic peaks, rebound after the end of treatment, or variation across the interdialytic interval. As a result, our data describe the direction and magnitude of change under routine HD conditions rather than detailed time–concentration trajectories. This tradeoff was chosen to ensure feasibility in a real-world dialysis setting and to minimize additional burden for patients, but more intensive serial sampling protocols will be needed in future studies to define the dynamic behavior of these peptide systems in greater detail. Another limitation is the heterogeneity of dialysis modalities, as both low-flux and high-flux membranes as well as haemodiafiltration were used according to routine clinical indications. Differences in membrane permeability and the presence of convective transport could theoretically influence peptide clearance. Although all treatments followed uniform adequacy targets within the same dialysis unit, the study was not designed or powered to support modality-specific comparisons. As such, potential effects of dialyzer type cannot be excluded and should be addressed in future studies employing predefined modality-stratified designs. Finally, because the primary aim was mechanistic rather than prognostic, cardiovascular outcomes were not evaluated. Future studies with larger cohorts, serial sampling across the full interdialytic cycle, detailed intradialytic monitoring, and isoform-specific assays will be needed to clarify the clinical and biomarker relevance of these peptide systems.

## Data Availability

Data are available from the corresponding author upon reasonable request.
